# Genomic Deletion at *10q23* in Prostate Cancer: More Than *PTEN* Loss?

**DOI:** 10.3389/fonc.2018.00246

**Published:** 2018-06-29

**Authors:** Raghavendra Tejo Karthik Poluri, Étienne Audet-Walsh

**Affiliations:** ^1^Department of Molecular Medicine, Axe Endocrinologie – Néphrologie du Centre de recherche du CHU de Québec, Université Laval, Québec, QC, Canada; ^2^Centre de recherche sur le cancer de l’Université Laval, Québec, QC, Canada

**Keywords:** steroid, androgen, castration-resistant, androgen receptor, neuroendocrine, androgen deprivation therapy

## Abstract

The *PTEN* gene encodes for the phosphatase and tensin homolog; it is a tumor suppressor gene that is among the most frequently inactivated genes throughout the human cancer spectrum. The most recent sequencing approaches have allowed the identification of *PTEN* genomic alterations, including deletion, mutation, or rearrangement in about 50% of prostate cancer (PCa) cases. It appears that mechanisms leading to *PTEN* inactivation are cancer-specific, comprising gene mutations, small insertions/deletions, copy number alterations (CNAs), promoter hypermethylation, and RNA interference. The examination of publicly available results from deep-sequencing studies of various cancers showed that PCa appears to be the only cancer in which *PTEN* is lost mostly through CNA. Instead of inactivating mutations, which are seen in other cancers, deletion of the *10q23* locus is the most common form of *PTEN* inactivation in PCa. By investigating the minimal deleted region at *10q23*, several other genes appear to be lost simultaneously with *PTEN*. Expression data indicate that, like *PTEN*, these genes are also downregulated upon loss of *10q23*. These analyses raise the possibility that 10q23 is lost upon selective pressure not only to inactivate *PTEN* but also to impair the expression of surrounding genes. As such, several genes from this deleted region, which represents about 500 kb, may also act as tumor suppressors in PCa, requiring further studies on their respective functions in that context.

## Introduction

The *PTEN* gene on chromosome *10q23* encodes for the phosphatase and tensin homolog, a tumor suppressor gene that is among the most frequently inactivated genes throughout the human cancer spectrum. Its lipid phosphatase activity allows PTEN to dephosphorylate phosphatidylinositol-triphosphate, therefore repressing the oncogenic PI3K/Akt/mTOR pathway. In prostate cancer (PCa), *PTEN* is frequently lost by deletion of the *10q23* region in tumors, which has been described several years ago (1–4), and prostate-specific deletion of *Pten* in mice leads to PCa development (5). The most recent sequencing approaches have allowed the identification of several types of genomic alterations of *PTEN*, including deletion, mutation, or rearrangement (including genomic inversions), and have further described *PTEN* alterations in about 50% of all PCa samples (6–14).

The inactivation rate of *PTEN* in PCa is similar to what has been described in other types of cancer, such as breast and endometrial cancers (15, 16). However, mechanisms leading to *PTEN* inactivation appear to be cancer-specific, comprising gene mutations, small insertions/deletions, copy number alterations (CNAs), promoter hypermethylation, and RNA interference (RNAi) (6–15). For example, endometrial cancer is characterized by microsatellite instability that is associated with frameshift mutations, which are the most frequent inactivating alterations in *PTEN* in that type of cancer (15, 17). In addition, patients with Cowden syndrome, who have a germline mutation in *PTEN*, also harbor a significantly higher risk of endometrial cancer (15, 18). Cowden syndrome is a rare autosomal-dominant condition that leads to an increased risk of breast, thyroid, and endometrial cancers (15, 19). However, patients with Cowden syndrome do not have increased risk of PCa, even though the loss of *PTEN* can be detected early in PCa patients (1, 6, 9, 13). It is reported that in PCa, between 2 and 15% of primary tumors harbor a *PTEN* mutation, while between 30 and 40% exhibit an important deletion on chromosome 10q23 (6, 10–13).

Importantly, many other genes are also present on the deleted region on *10q23*, most of which have not been investigated in the context of PCa. Moreover, because *PTEN* is more frequently deleted in PCa through large genomic deletions and at a higher frequency compared with other types of cancer instead of the gene-specific mutations that occur in most malignancies, it raises the possibility that genes lost at the same times as *PTEN* in PCa also display important tumor suppressor functions. It is well accepted now that large genomic deletions can contain more than one important gene, but this concept was not investigated in the context of the loss of *10q23* in PCa. In this perspective article, we will discuss the genes that are lost along with *PTEN* upon deletion of the *10q23* locus that might well play a role in PCa development.

## *PTEN* is More Frequently Altered through CNA Rather than Through a Specific Gene Mutation in PCa

**Figure 1:**
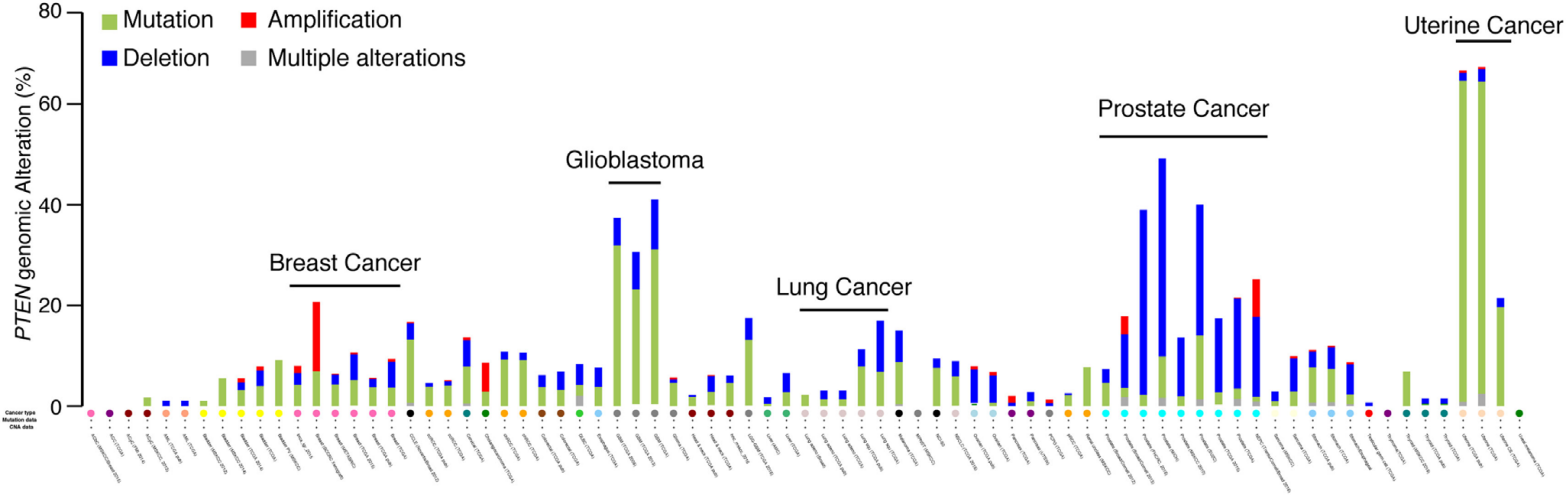
*PTEN* genomic alterations in the human cancer spectrum. Genomic alterations of the *PTEN* gene were visualized with the cBioPortal for Cancer Genomics (20, 21). Only cohorts with data on both mutation and copy number alterations are shown.

The *PTEN* genomic status was first screened through the cancer spectrum across the different cohorts available on the cBio Cancer Genomics Portal from *The Cancer Genome Atlas* (TCGA) group (20, 21). Only cohorts with both mutation and CNA were kept for analysis. In most types of cancer, *PTEN* is often mutated (Figure 1, green color), with particularly high mutations rates in glioblastoma and uterine cancer, where the alterations rate is between 40 and 65% (Figure 1). Despite showing small CNA rates, gene mutations are the most common *PTEN* genomic alterations. Interestingly, the only type of cancer with high rates of *PTEN* genomic alterations particularly caused by CNA is PCa. In PCa, between 20 and 50% of all tumors exhibit *PTEN* alterations, with 60 and 90% of them being CNA instead of mutations (Figure 1). This suggests that CNA at the *PTEN* locus might affect more than *PTEN* itself and lead to the deletion of other tumor suppressor genes important to the etiology of PCa.

**Figure 2:**
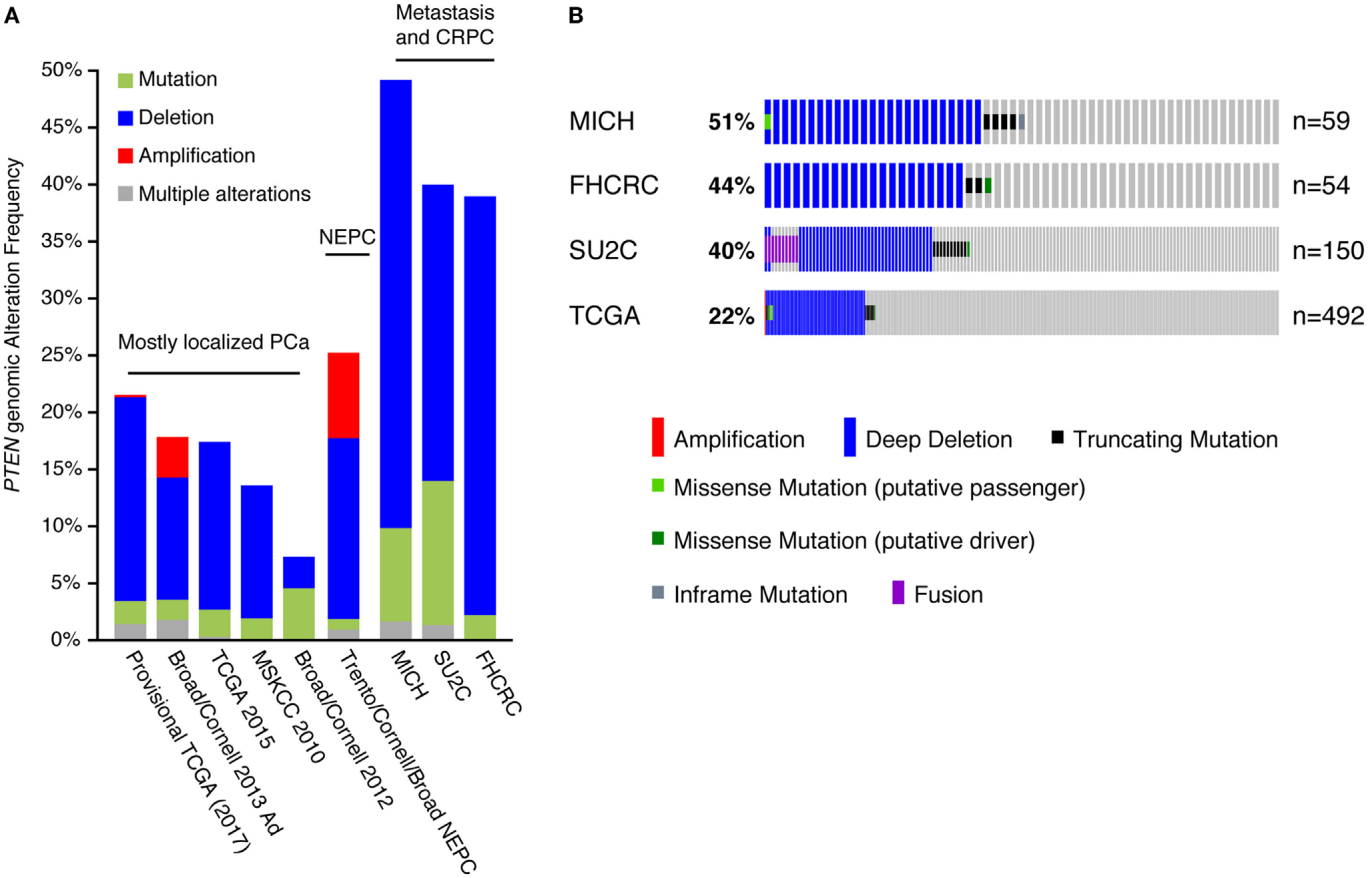
Copy number alteration (CNA) is the most frequent inactivation mechanism of *PTEN* in human prostate cancer (PCa). **(A)** Genomic alterations of the *PTEN* gene in PCa. Only cohorts with data on both mutation and CNAs are shown. Abbreviations: NEPC, neuroendocrine prostate cancer; CRPC, castration-resistant PCa. **(B)** Specific alterations of *PTEN* in the Michigan (MICH), Fred Hutchinson Cancer Research Center (FHCRC), Stand Up To Cancer (SU2C), and *The Cancer Genome Atlas* (TCGA) cohorts. Note that the proportion of alterations is slightly different than in **(A)**: all tumors are shown in **(A)** while all patients are shown in **(B)** (some patients had more than one sample sequenced).

We further analyzed the *PTEN* genomic alteration status between the different PCa cohorts (Figure 2A), which included the Michigan, Stand Up To Cancer (SU2C), and Fred Hutchinson Cancer Research Center datasets, mostly comprising metastatic samples (12, 13, 22); the Trento/Cornell/Broad dataset, composed of metastatic neuroendocrine prostate cancer (23); the Broad/Cornell 2012, Broad/Cornell 2013, Memorial Sloan Kettering Cancer Center, and the two TCGA datasets, comprising mostly primary localized PCa (9, 11, 16, 24). In cohorts mostly composed of clinically localized tumors, *PTEN* genomic alterations, mostly CNA, ranged from 10 to 20%. This alteration frequency increased to 40–50% in cohorts of metastatic samples and castration-resistant PCa (CRPC) tumors, again mostly through CNA of *PTEN*. These results are consistent with previous reports linking *PTEN* loss to PCa aggressiveness as it is increased in more aggressive disease settings (25–28). Further investigation of *PTEN* alterations in the CRPC/metastatic cohorts confirmed higher rates of CNA in these tumors compared with the TCGA cohort, which is mostly composed of clinically localized tumors (Figure 2B). These data also confirmed that deletion of *PTEN* is the most frequent genomic alteration occurring at this locus in prostate tumors.

## CNA at *10q23* Leads to Loss not only of *PTEN* but also of Several Additional Genes

**Figure 3:**
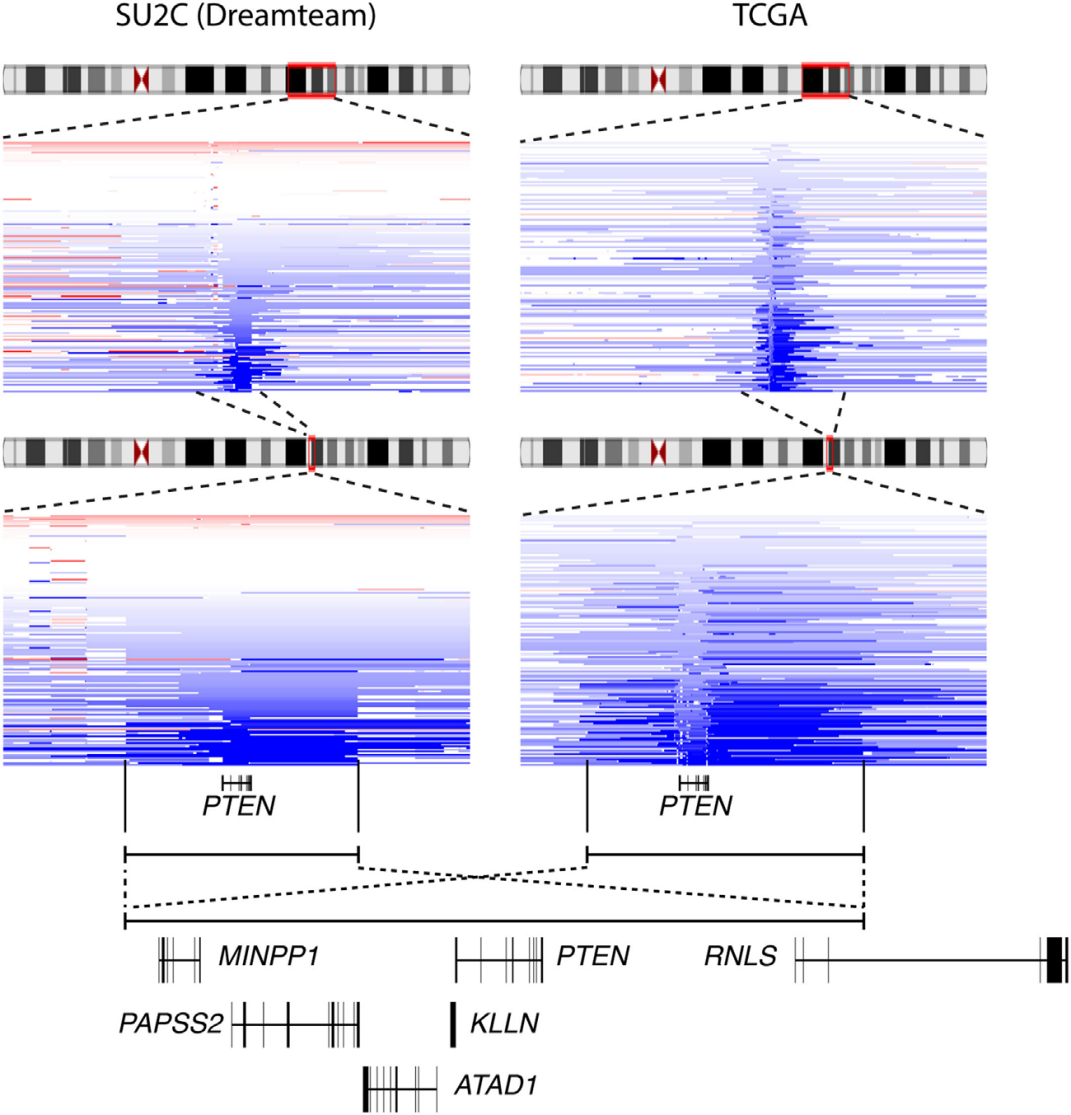
The minimal deletion at *10q23* in prostate cancer (PCa). Deletion status at the *10q23* locus in the Stand Up To Cancer (SU2C) (left) and the *The Cancer Genome Atlas* (TCGA) (right) PCa clinical datasets. Blue color indicates copy number loss at this locus. Genes present in that genomic regions are shown (bottom panel).

**Figure 4:**
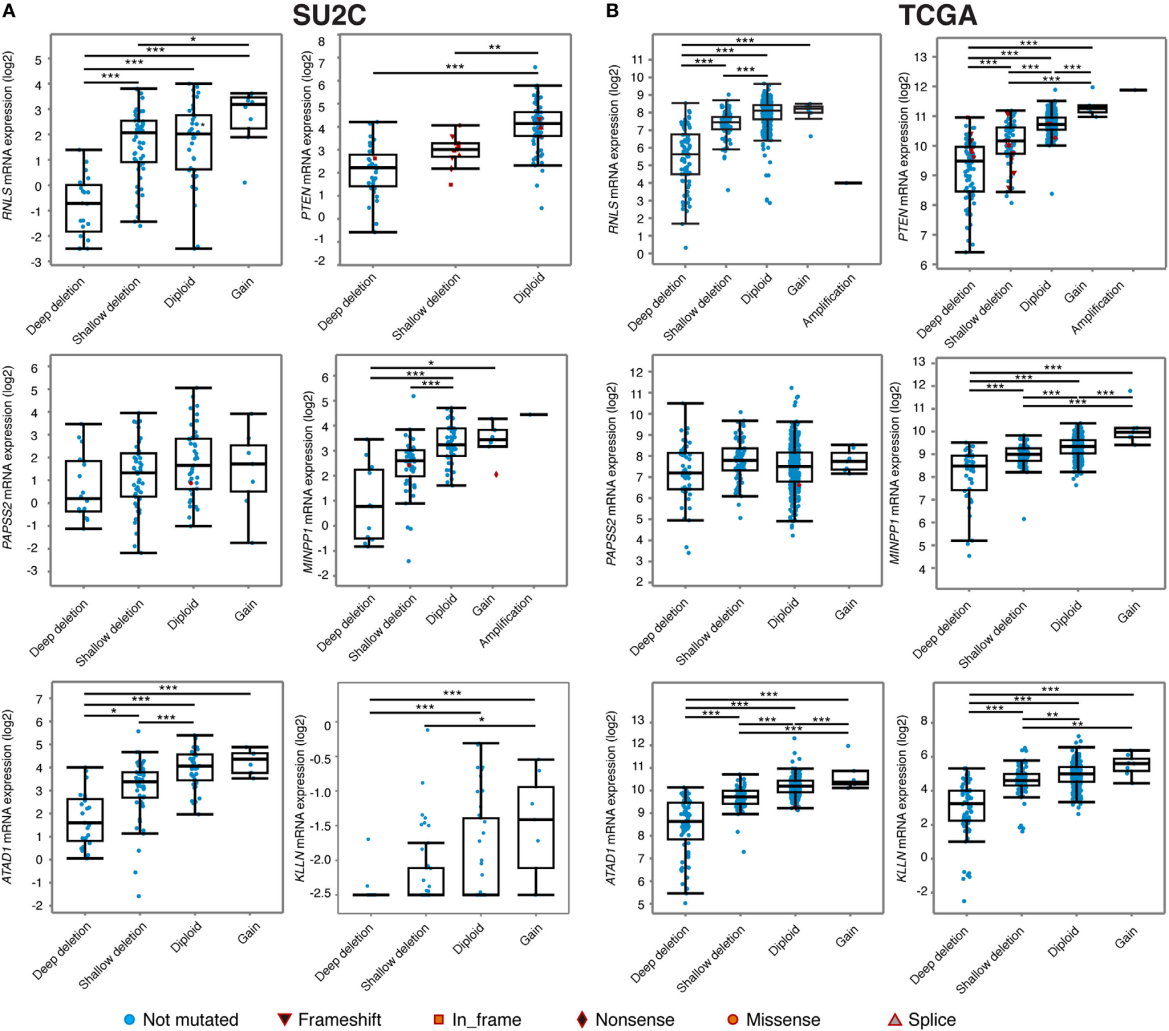
Relationship between genomic alterations at the *10q23* locus and genes encompassed within this region. Expression of six genes located at the minimal deleted regions at the *10q23* locus in the Stand Up To Cancer (SU2C) **(A)** and the *The Cancer Genome Atlas* (TCGA) **(B)** prostate cancer cohorts. ***p < 0.001; **p < 0.01; *p < 0.05 in ANOVA with *post hoc* Tukey’s Honest Significant Difference. Note that for *RNLS* and *PTEN* expression data in the TCGA cohort **(B)**, the sample with an amplification at *10q23* was not included for statistics.

Interestingly, visualization of CNA at the *PTEN* locus in the various cohorts available on the cBioportal indicated that deletion of *PTEN* often results in the loss of a large (>500 kb) genomic segment of chromosome *10q23* (Figure 3). Results from the metastatic cohort SU2C revealed that *PTEN* is commonly lost with other genes located at *10q23*, including *MINPP1, PAPSS2, KLLN*, and *ATAD1*. Moreover, deletion at *10q23* frequently occurs in one of the *RNLS* introns. By also investigating other cohorts with high coverage at *10q23* CNA status, we observed a similar deletion pattern, notably in the localized PCa cohort from the provisional TCGA dataset (Figure 3, right). Again, the same similar minimal region seems to accompany the loss of *PTEN*, altering the same set of genes as in the more aggressive SU2C cohort, including the deletion breakpoint in the intronic region of *RNLS*. These results suggest that the loss of *10q23* in PCa cells does not solely inactivate the tumor suppressor *PTEN*, but that there is also a selective pressure to lose other gene(s) at this particular genomic region in this specific type of cancer. Accordingly, CNA at *10q23* significantly altered mRNA expression of *PTEN*, and deletions of *PTEN* resulted in decreased mRNA expression in both the SU2C and the TCGA cohorts (Figures 4A,B, respectively). Genes surrounding *PTEN* that are located in the minimal deleted regions (Figure 3) also have a similar pattern, with a significantly decreased expression with either shallow or deep deletions in the two cohorts (Figure 4). The only exception was *PAPSS2*, which was not significantly altered by deep or shallow deletion (Figure 4). As reported previously (13, 16), various inactivating mutations of *PTEN* were also detected in both cohorts, but at lower frequency than CNA.

## Future Direction in PCa Genomic Alteration Studies

Since the initial characterization of *PTEN* loss in PCa two decades ago (1–4), most studies on CNA at *10q23* have focused exclusively on *PTEN* as basically the only gene lost upon deletion at this specific locus, even in more recent deep-sequencing studies (6–14). Given its known role as a tumor suppressor in most types of cancer and because mutation of *PTEN* causes a hereditary syndrome with multiple cancer susceptibilities, *PTEN* is most certainly the main tumor suppressor gene lost with this deletion. However, other genes located in the minimal deleted region at *10q23* might play a significant role in PCa etiology because (1) CNA is the major genomic alteration in PCa, not a direct mutation of *PTEN*, which occurs in all other types of cancer; (2) a large region at *10q23* is deleted, comprising more than just *PTEN* and often including the loss of at least six other genes; and (3) some of these genes have already been associated with tumor suppressing functions in PCa or other cancers. The question remains as to what roles these genes play in prostate biology and PCa development.

*KLLN* encodes for the KILLIN protein, which has been identified as a P53 target required for S phase checkpoint control to eliminate precancerous cells (29). *KLLN* overexpression reduces PCa cell growth *in vitro* by decreasing the androgen receptor (AR) signaling, while its repression increases it; this is consistent with a tumor suppressor function of this gene (30). Interestingly, mutation in the promoter of *KLLN* is also associated with Cowden and Cowden-like syndromes, possibly by sharing its promoter with *PTEN* itself (31). *ATAD1* depletion induces mitochondrial fragmentation and impairs respiration (32). It is notable that increased mitochondrial respiration is a key metabolic phenotype associated with PCa development and progression (33–37). *RNLS* encodes the renalase FAD-dependent metabolic enzyme (38), which has no currently known role in PCa. *PAPSS2* encodes for PAPS synthase 2, which provides sulfate donors to sulfotransferase enzymes, including SULT2A1, which is a critical enzyme for dehydroepiandrosterone (DHEA) sulfation (39). DHEA and its sulfate form (DHEA-S) represent the major adrenal androgen precursors and therefore are important sources for intra-tumor androgen synthesis. This is particularly relevant during PCa progression and is a therapeutic target used in the clinic (adrenal androgens production is inhibited using abiraterone acetate) (40–42). The few patients identified with mutations in this gene are female, and heterozygous inactivation of *PAPSS2* has been associated with polycystic ovary syndrome, premature puberty, hyperandrogenic anovulation, very low DHEA-S levels, and increased androgen levels (43). Even though *PAPSS2* is not significantly decreased by CNA, intra-tumor loss of *PAPSS2* could favor androgen excess and hyperactivation of AR, which is critical for tumor growth and cancer progression. *MINPP1* encodes a phosphatase linked to inositol-3-phosphate metabolism, similar to PTEN functions (44). *CFL1P1* is cofilin pseudogene 1 and has no known function. However, based on mRNA expression profiles across tissues from the data presented by Fagerberg et al. (45), it shows high expression specifically in the testis, possibly reflecting a function in the male reproductive system.

Further experiments are now required to characterize the role of these genes located near *PTEN* and lost along with this important tumor suppressor in PCa. Only a few *in vitro* models of human PCa exist, some that exhibit complete loss of *PTEN*, such as in PC3 cells; some that exhibit mutation of *PTEN* and partial loss at *10q23*, such as LNCaP cells; and finally some that harbor wild-type *PTEN*, such as 22rv1 and DU145 cells (4, 9, 46). These wild-type PCa cell lines thus represent potential *in vitro* models to study the impact on PCa cell proliferation of *PTEN* inactivation, with and without inactivation of one or more of the other genes located within the minimal deleted region at *10q23* (*KLLN, ATAD1, RNLS, PAPSS2, MINPP1*, and *CFLIP1*). Because in most tumors there is a single copy lost at *10q23* in most tumors, and not a complete loss of both copies, repression of these genes with RNAi would mimic the gene expression decrease observed in tumor samples. The more recent genome editing technology using the CRISPR/Cas9 system would also allow the knockout of these genes along with *PTEN* to study their potential role as tumor suppressors of PCa *in vitro* (47).

Interestingly, the mouse genome exhibits a similar gene architecture around *Pten*, with at least the presence of *Atad1, Rnls, Papss2*, and *Minpp1*. The prostate-specific loss of *Pten* PCa mouse model (*Pten*^flox/flox^;PB-Cre4^+^) is one of the most commonly used *in vivo* models to study PCa development and progression (5, 48). In this model, *Pten* is inactivated by deletion of exon 5 (49) and other genes surrounding *Pten* are not altered. The single knockout of these genes in the *Pten*^flox/flox^;PB-Cre4^+^ would allow the study of their function as tumor suppressors in this PCa mouse model *in vivo*. In addition, the development of a new PCa mouse model by inducing the loss of *19qC1*, the loci that contains *Pten* in the mouse genome, would mimic the human loss of *10q23* and would be highly informative on the biological functions as tumors suppressors of this region in comparison to the disruption of *Pten* only. This type of genetic engineering approach was successfully used to study oncogenic chromosomal rearrangements in mouse models of human cancers, such as the *EML4-ALK* oncogene in lung cancer (50). Indeed, induction of this gene fusion in adult mice promotes the development of non-small-cell lung cancers, clearly demonstrating the oncogenic properties of this chromosomal rearrangement. Inducing the loss of *19qC1* followed by rescue of *Atad1, Rnls, Papss2*, and *Minpp1* would also be an interesting approach to study the role of all the genes lost along with *PTEN* in human and their impact on PCa development and progression.

## Concluding Remarks

In summary, using publicly available results from deep-sequencing studies of various cancers, PCa appears to be the only cancer in which *PTEN* is inactivated mostly through CNA. Large genomic deletions often contain more than one important gene, and this is a concept that needs to be revisited in the context of *PTEN* loss in human PCa. Instead of inactivating mutations as seen in other cancers, deletion at *10q23* is the most common form of *PTEN* inactivation. Investigation of the minimal deleted region at *10q23* revealed that several other genes appear to be lost in addition to *PTEN*. Expression data indicate that, like *PTEN*, these genes are downregulated upon CNA, and, together with the CNA profile, suggest that these genes represent potential novel tumor suppressor genes in PCa. Their potential function as PCa tumor suppressors thus remained to be determined using state-of-the-art genetic engineering approaches in *in vitro* and *in vivo* models of PCa.

## Author Notes

Data are publicly available through the TCGA cBioPortal web platform for cancer genomics. All ethics approvals and consents to publish were obtained for the original studies.

## Author Contributions

EAW conceptualized the study and performed the analyses. RTKP and EAW analyzed the data and wrote the manuscript.

## Conflict of Interest Statement

There are no competing financial interests to disclose. Data are publicly available through the TCGA cBioPortal web platform for cancer genomics. All ethics approvals and consents to publish were obtained for the original studies. The authors declare that the research was conducted in the absence of any commercial or financial relationships that could be construed as a potential conflict of interest.
